# Association of hyperventilation-induced heart rate variability and sudden unexpected death in epilepsy in drug-resistant epilepsy

**DOI:** 10.1055/s-0044-1791517

**Published:** 2024-11-03

**Authors:** Demet Ilhan Algin, Oguz Erdinc

**Affiliations:** 1 Eskisehir Osmangazi University Faculty of Medicine, Department of Neurology, Eskisehir, Turkey.

**Keywords:** Sudden Unexpected Death in Epilepsy, Epilepsy, Hyperventilation, Morte Súbita Inesperada na Epilepsia, Epilepsia, Hiperventilação

## Abstract

**Background** Within the general epilepsy population, the incidence of Sudden Unexpected Death in Epilepsy (SUDEP) ranges from approximately 0.35 to 2.3 per 1,000 individuals per year.

**Objective** We aimed to evaluate the relationship between SUDEP risk factors and heart rate variability (HRV) parameters as a potential biomarker of SUDEP in patients with drug-resistant epilepsy (DRE).

**Methods** There were 52 patients diagnosed with DRE and under follow-up, and controls including 45 healthy subjects, included in the study. Hyperventilation-induced HRV (HRV _HV_ ) parameters, including the standard deviation of all RR intervals (SDRR), mean heart rate (HR), root mean squares of successive differences (RMSSD), SD of mean NN intervals recordings (SDANN), and HRV triangular index, were assessed during resting. To predict the risk of SUDEP, the relationship between HRV parameters and SUDEP risks was evaluated using the Risk Assessment for Sudden Death in Epilepsy (SUDEP-7) Risk Inventory.

**Results** No statistically significant difference was found in sympathetic skin response (SSR) latency and amplitudes between the patient and control groups. In comparing healthy control subjects with patients experiencing DRE, we observed significant decreases in SDRR _HV_ and hyperventilation-induced RMSSD (RMSSD _HV_ ) values, specifically within HRV _HV_ . Notably, a significant correlation emerged concerning the RMSSD _HV_ values ( *p* < 0.01), when examining the correlation between the SUDEP-7 inventory and HRV _HV_ parameters.

**Conclusion** This correlation between RMSSD _HV_ and the SUDEP-7 Risk Inventory in patients with DRE represents a novel and consequential finding, suggesting its potential as an indicator of SUDEP risk.

## Resumo

**Antecedentes** Na população geral com epilepsia, a incidência de morte súbita inesperada na epilepsia (SUDEP) varia de aproximadamente 0,35 a 2,3 por 1.000 indivíduos por ano.

**Objetivo** Nosso objetivo foi avaliar a relação entre os fatores de risco de SUDEP e os parâmetros de variabilidade da frequência cardíaca (VFC) como um possível biomarcador de SUDEP em pacientes com epilepsia refratária (ER).

**Métodos** O estudo incluiu 52 pacientes diagnosticados com ER e em acompanhamento e 45 indivíduos saudáveis como controles. Os parâmetros de VFC induzidos por hiperventilação (VFC _HV_ ), incluindo o desvio padrão de todos os intervalos RR (SDRR), frequência cardíaca (FC) média, raiz quadrada média das diferenças sucessivas (RMSSD), DP dos registros de intervalos NN médios (SDANN) e índice triangular de VFC, foram avaliados durante o repouso. Para prever o risco de SUDEP, a relação entre os parâmetros de VFC e os riscos de SUDEP foi avaliada usando o Inventário de Avaliação de Risco de Morte Súbita em Epilepsia ( *Risk Assessment for Sudden Death in Epilepsy* , SUDEP-7).

**Resultados** Não houve diferença estatisticamente significativa na latência e amplitudes da resposta simpática da pele (SSR) entre os grupos de pacientes e controles. Ao comparar indivíduos controles saudáveis a pacientes com ER, observamos reduções significativas nos valores de SDRR induzido por hiperventilação (SDRR _HV_ ) e RMSSD induzida por hiperventilação (RMSSD _HV_ ), especificamente dentro de VFC _HV_ . Houve uma notável correlação significativa em relação aos valores de RMSSD _HV_ (p < 0,01) entre o inventário SUDEP-7 e os parâmetros de VFC _HV_ .

**Conclusão** Essa correlação entre RMSSD _HV_ e o Inventário de Risco SUDEP-7 em pacientes com ER representa um achado novo e consequente, sugerindo seu potencial como um indicador de risco de SUDEP.

## INTRODUCTION

Among the general epilepsy population, the incidence of sudden unexpected death in epilepsy (SUDEP) ranges from approximately 0.35 to 2.3 per 1,000 individuals per year. 1 2 Notably, patients grappling with drug-resistant epilepsy (DRE), especially those undergoing epilepsy surgery or continuing to experience seizures postsurgery, face a heightened risk of SUDEP, with an incidence rate ranging from 6.3 to 9.3 per 1,000 individuals per year. 1 The intricate pathophysiological mechanisms underlying SUDEP involve multifaceted factors, including complex alterations in the sympathetic and parasympathetic systems during seizures. Contributions from cardiac, respiratory, and metabolic changes, and circulatory disorders either independently or synergistically contribute to the onset of SUDEP. In this context, the SUDEP-7 Risk Inventory was improved as a clinical SUDEP risk marker and, since then, it has been used to assess the potential SUDEP biomarkers. 3

Studies involving healthy individuals have disclosed that both hypercapnia and hypoxemia can impact cardiac repolarization, tilting the autonomic balance toward heightened sympathetic activity in the sinus node. 4 5 To give a few examples, Seyal et al. 6 discovered a positive correlation between the extent of cardiac repolarization abnormalities and ictal oxygen desaturation level in epilepsy patients, and the SUDEP case study performed within the scope of the Mortality in Epilepsy Monitoring Unit Study (MORTEMUS) exhibited a complex and eventually fatal cardiopulmonary dysfunction which has emerged in the early stages of the seizure. 7

Epilepsy patients commonly exhibit interictal autonomic dysfunction and reduced heart rate variability (HRV), as observed in several studies. During hyperventilation (HV), incremental hypocarbia and hypoxia may provoke fatal arrhythmias through chronic and repeated activation of the autonomic nervous system, fostering a sympathovagal imbalance. 4 5

Heart rate variability parameters may not be always atypical at rest. Therefore, they can be induced by response to physiological stress. An increase in heart rate against mental or physical stress reflects the function of the autonomic nervous system. The heart rate response to stress is usually assessed by exercise or pharmacological injection. Hyperventilation is a self-controlled respiratory maneuver, and it has been revealed to have diagnostic and prognostic capasity to assess heart rate changes. Currently, HV has been used as a tool to research the myocardial oxygenation and heart disease in the cardiovascular magnetic resonance (CMR) studies. 5

Given the aforementioned considerations, the present study aims to assess the relationship between sympathetic skin responses, HRV parameters at rest, and after HV with SUDEP risk in patients with DRE. The evaluation will be conducted using the SUDEP-7 Risk Inventory.

## METHODS

Fifty-two patients undergoing follow-up with a diagnosis of DRE at the epilepsy outpatient clinic, along with 45 healthy controls, were included in the study. We performed this study in accordance with the Ethical Standards of the Declaration of Helsinki. Ethical committee approval for the study was obtained from the Ethical Committee of Eskisehir Osmangazi University Non-Interventional Clinical Research (Date: Feb 28, 2020, No: 2019–279). Additionally, a signed written informed consent form was obtained from all participants in the study.

Patients with DRE were selected based on the 2010 definition provided by the International League Against Epilepsy (ILAE). 8 Detailed anamnesis was collected for all cases in the patient group, and physical and neurological examinations were performed. Individuals with evident neurologic risk factors, such as alcoholism, diabetes, vitamin B12 deficiency, exposure to neurotoxic drugs, thyroid dysfunction, or renal failure, were excluded from the study. Before assessing the autonomic nervous systems of patients and healthy controls in the study, all participants underwent electroneuromyographic analysis to investigate them for polyneuropathy. Consequently, individuals determined to have polyneuropathy were excluded from this study.

Demographic characteristics of the patients, etiology information, age of epilepsy onset, duration of epilepsy, relevant medications, responses to administered treatments, and EEG and neuroimaging findings were recorded.

### Sympathetic skin response and HRV measurement

The electrophysiological examination protocol for autonomic nervous system (ANS) functions involved the assessment of the sympathetic nervous system through sympathetic skin response (SSR) and the parasympathetic nervous system through heHRV, measured both at rest and during deep breathing.

Sympathetic skin response values obtained from both upper extremities of both patients and control subjects, along with RR interval variation (RRIV) values recorded both at rest and during deep breathing using a Medtronic electromyography (EMG) device, were scrutinized. Sympathetic skin response tests were conducted on both upper limbs. The active electrode was positioned on the right palm, and the reference electrode was placed on the dorsum of the hand. Electrical stimulation, ranging from 20 to 100 mA, was applied over the median nerve of the left wrist. For SSR latency, the duration from the onset of the stimulus artifact to the initiation of the first deflection (typically negative) was measured.

During the assessment of the HRV, an active reference electrode was placed on the dorsum of the right hand and a reference electrode was placed on the dorsum of the left hand. The first measurement was performed while the patient or control subject was resting in a sitting position, and the subsequent measurements were performed during deep breathing every 10 seconds, that is, 5 seconds for inspiration plus another 5 seconds for expiration, and 6 times a minute. In this method, filters are selected as 5 to 100 Hz, trigger mode is used, and the sweep is triggered by each ECG complex. The “event pulse” generated synchronously with each trigger potential is sent to the internal clock of the computer. The time difference between successive pulses is measured, and standard deviation of all RR intervals (SDRR), hyperventilation-induced mean heart rate (HR), root mean squares of successive differences (RMSSD), standard deviation of mean NN intervals (SDANN) recordings, and heart rate variability triangular index (HRV-TI) values were determined during the rest period. This assessment was conducted within a 1-minute time interval using specialized software installed on the device.

### Statistical methods

Nominal parameters were characterized using frequencies, while scale parameters were described using means and standard deviations. The normality of scale parameters was evaluated using the Kolmogorov-Smirnov Test. For normally distributed scale parameter differences, Independent Samples *t* -test and Paired Samples *t* -test were applied. The Mann-Whitney U- and Wilcoxon signed-rank tests were utilized for non-normally distributed parameter differences. All analyses were conducted using the SPSS Statistics for Windows, v. 17.0 (SPSS Inc., Chicago, IL, USA) with a confidence interval of 95%.

## RESULTS

Fifty-two patients (27 males and 25 females) with DRE, and 45 age- and sex-matched healthy controls (23 males and 22 females) were enrolled in the study. The mean age of patients and healthy control subjects was 35.36 ± 13.90 and 39.10 ± 8.11 years, respectively. The mean duration of epilepsy was 18.10 ± 12.03 years, and the mean seizure frequency was 8.32 ± 2.64 attacks per month. Statistically significant difference was not observed between the SDR latency and amplitude values in both groups ( Table 1 ). Among the patients, 41 (78.9%) had temporal lobe epilepsy, 6 (11.5%) had frontal lobe epilepsy, 4 (9.6%) had generalized epilepsy, and 1 patient (1.9%) had myoclonic epilepsy with ragged-red fibers (MERF).

**Table 1:** Baseline characteristics of patient groups and controls

		Patient ( *n* = 52)	Control ( *n* = 45)	*p*
Gender, n (%)	Female	25 (48.1)	22 (46.6)	0.967 ^a^
	Male	27 (51.9)	23 (53.3)	
Age, mean ± SD		35.36 ± 13.90	39.10 ± 8.11	0.162 ^b^
Epilepsy duration, mean ± SD		18.10 ± 12.03	−	N/A
Seizure frequency, mean ± SD		8.32 ± 2.64	−	N/A
SUDEP-S, mean ± SD		4.05 ± 2.45	−	N/A
Anti-epileptic drugs (AEDs), mean ± SD		2.85 ± 0.82	−	N/A
SDR Latency, mean ± SD		1,584.12 ± 262.09	1,691.9 ± 370.32	0.565 ^c^
SSR Amplitude, mean ± SD		1.45 ± 1.22	1.56 ± 1.25	0.802 ^c^

Abbreviations: N/A, not applicable; SD, standard deviation; SSR, sympathetic skin response.

Notes: ^a^ Chi-squared test. ^b^ Independent Samples *t* -test. ^c^ Mann-Whitney U test.

Gender, age, and SSR amplitude parameter differences were not significant between the patient groups ( *p* > 0.05).

Out of the 52 patients undergoing follow-up with a diagnosis of DRE, 39 (75%) had a structural etiology, 4 (7.7%) had a genetic etiology, 3 (5.8%) had an infectious etiology, and 2 (3.8%) had an immune etiology. The etiology in 4 (7.7%) patients remained unknown. Among the cases with a structural origin, epilepsy was attributed to mesial temporal sclerosis (33.3%), cortical dysplasia/heterotopia (15.4%), cerebrovascular/degenerative diseases (20.5%), brain tumors (15.4%), cavernous angioma (10.2%), and trauma (5.1%). Etiological classification adhered to the ILAE 2018 classification 9 ( Table 2 ).

**Table 2:** Etiology of drug-resistant epilepsy patients

	N = 52
Structural	39 (75%)
Mesial temporal sclerosis	13 (33.3%)
Cortical dysplasia/heterotopia	6 (15.4%)
Cerebrovascular/dejenerative diseases	8 (20.5%)
Brain tumor	6 (15.4%)
Cavernous angioma	4 (10.2%)
Trauma	2 (5.1%)
Genetic	4 (7.7%)
Infectious	3 (5.8%)
Immune	2 (3.8%)
Unknown	4 (7.7%)

Resting and hyperventilation-induced HRV _HV_ parameter difference analysis results are shown in Table 3 .

**Table 3:** Resting and hyperventilation-induced ( _HV_ ) HRV parameter difference analysis results

		Study ( *n* = 52)	Control ( *n* = 45)	*p*
*Resting HRV*	HR	78.26 ± 10.92	74.78 ± 11.32	0.200 ^a^
	Max-min mean HR	27.97 ± 23.25	26.32 ± 16.78	0.948 ^b^
	SDRR	0.036 ± 0.02	0.042 ± 0.02	0.591 ^b^
	RMSSD	0.047 ± 0.03	0.129 ± 0.02	0.043 ^b^
	HRV-TI	5.89 ± 2.67	6.64 ± 2.27	0.220 ^a^
* Hyperventilation-induced ( _HV_ )HRV *	HR _HV_	85.08 ± 13.40	88.38 ± 10.81	0.223 ^a^
	Max-min mean HR _HV_	40.92 ± 27.83	38.67 ± 18.17	0.779 ^b^
	SDRR _HV_	0.062 ± 0.03	0.134 ± 0.03	0.047 ^a^
	RMSSD _HV_	0.038 ± 0.06	0.142 ± 0.03	0.036 ^b^
	HRV-TI _HV_	8.89 ± 3.37	9.35 ± 2.47	0.527 ^a^

Abbreviations: HR, heart rate; HR ^HV^ , hyperventilation-induced heart rate; HRV-TI, heart rate variability triangular index; HRV TI _HV_ , hyperventilation-induced heart rate variability heart rate variability triangular index; min, minimum; max, maximum; N/A, not applicable; RMSSD, root mean squares of successive differences; RMSSD _HV_ , hyperventilation-induced root mean squares of successive differences; SD, standard deviation; SDRR, standard deviation of all RR intervals; SDRR _HV_ , hyperventilation-induced standard deviation of all RR intervals.

Notes: ^a^ Independent Samples *t* -test. ^b^ Mann-Whitney U-test.

Resting and hyperventilation-induced RMSDD (RMSDD _HV_ ) as well as SDRR _HV_ level differences were significant between patient and control groups ( *p* < 0.05). The results of resting and hyperventilation-induced (HV) HRV parameter difference analysis are shown in Table 3 .

Correlations between SUDEP score and HRV parameters are shown in Table 4 .

**Table 4:** Spearman correlation between HRV parameters and SUDEP-7 scores

HRV parameters	r	*p*
HR	0.265	> 0.01
HR _HV_	0.184	> 0.01
Max-min mean HR	0.151	> 0.01
Max-min mean HR _HV_	0.213	> 0.01
SDRR	0.196	> 0.01
SDRR _HV_	0.317	> 0.01
RMSSD	0.578**	< 0.01
RMSSD _HV_	0.749**	< 0.01
HRV-TI	0.167	> 0.01
HRV-TI _HV_	0.192	> 0.01

Abbreviations: HR, heart rate; HR ^HV^ , hyperventilation-induced heart rate; HRV-TI, heart rate variability triangular index; HRV TI _HV_ , hyperventilation-induced heart rate variability heart rate variability triangular index; min, minimum; max, maximum; RMSSD, root mean squares of successive differences; RMSSD _HV_ , hyperventilation-induced root mean squares of successive differences; SDRR, standard deviation of all RR intervals; SDRR _HV_ , hyperventilation-induced standard deviation of all RR intervals.

Notes: ** *p* < 0.01 hyperventilation-induced ( _HV_ ). According to correlation analysis results, RMSSD (r = 0.578; *p* < 0.01) and RMSSD _HV_ ; (r = 749; *p* < 0.01) positively correlated with SUDEP score.

Among the HRV parameters, RMSSD (r = 0.578) and RMSSD _HV_ (r = 0.749) were significantly correlated with SUDEP-7 scores. Hyperventilation-induced RMSSD showed the strongest and most significant correlation. Root mean squares of successive differences was moderately correlated with SUDEP-7 scores.

## DISCUSSION

Our objective was to compare the resting and HV-induced variability of HRV parameters and correlation with SUDEP-7 score in DRE patients. The results indicate that RMSDD _HV_ values are more strongly correlated than the values at rest.

Increased HRV is an important indicator that the autonomic control mechanism functions well in healthy individuals, contrary to the decreased HRV, which is an important indicator of mortality.

Parasympathetic innervation is related to the increased HRV, whereas sympathetic innervation is associated with decreased HRV. Epilepsy patients frequently have interictal autonomic dysfunction, as well as reduced HRV, as reported by many studies. 10 Interictal HRV abnormalities have been shown in various epilepsy populations, including patient populations with focal (particularly temporal lobe epilepsy) and generalized epilepsy. 11 12 Furthermore, other types of autonomic tests, including baroreflex function and Valsalva maneuver tests, also demonstrate interictal dysfunction in epilepsy. 10

Decreased HRV is a significant predictor of sudden death in patients with heart disease. Heart rate variability is typically reduced in epileptics, particularly in cases with temporal lobe or drug-resistant seizures. 13 However, there is still not enough evidence that HRV, as a biomarker, 14 predicts the SUDEP risk, except for epilepsies with sodium channel mutations. 15 In a study conducted on 47 patients with DRE and 45 healthy control subjects, patients with epilepsy were determined to have decreased HRV, but the decrease in HRV could not be associated with the SUDEP-7 inventory score. 16 In another study, in which 39 patients with DRE and 33 healthy control subjects were compared in terms of the interictal heart rates and the associated 24-hour changes, abnormalities were detected in the patient group in respect of all parameters. Specifically, a significantly decreased heart rate was observed in patients between 3 and 5 a.m., a phenomenon attributed to the inhibition of vagal modulation. 17

In a study in which the intracranial EEG (iEEG) and electrocardiography (ECG) findings were investigated, 13 patients with refractory temporal lobe epilepsy were found to have a significantly higher heart rate during bitemporal ictal activity, and their HRV parameters were determined to have significantly decreased. The increase in heart rate and the decrease in HRV were attributed to the activity of the autonomic nervous system, specifically directed toward the sympathetic system. 18

There are many studies available in the literature, in which the HRV parameters in relation to SUDEP risk factors were studied in patients with epilepsy; nevertheless, the HRV data directly obtained from SUDEP patients are limited, and no clear HRV biomarkers specific to SUDEP could be determined until now. 19

More recently, in a study by Myers et al., epilepsy patients with sodium channel mutations were compared with patients with refractory epilepsy. It was observed that epilepsy patients with sodium channel mutations had lower HRV in the awake state. Additionally, the ratios of HRV values measured during the awake state to those measured during sleep were either excessively high or low in this particular group. 20

Ictal asystole (IA) appears to be a self-terminating event, yet it is not known whether a history of periictal bradycardia, asystole, or non-sustained ventricular tachycardia increases the risk of SUDEP. 21

In a study, potentially high cardiac arrhythmias were investigated in 69 patients with focal-to-bilateral tonic-clonic seizures (FBTCSs) and generalized tonic-clonic seizures (GTCSs). The study revealed a significant relation between potentially high-risk cardiac arrhythmias and the duration of ictal/postictal hypoxemia in 10 patients with FBTCS/GTCS. This finding was attributed to the fact that hypoxemia triggered fatal cardiac arrhythmias in patients with SUDEP. 22

Additionally, it has been suggested that the abnormal regulation of blood pressure during seizures may be a contributing factor to SUDEP. Furthermore, baroreflex sensitivity (BRS) was investigated in patients with epilepsy in two recent studies. 23 24 In the 1 ^st^ study, 14 patients with focal seizures and 9 patients with GTCS were investigated. Baroreflex sensitivity was found to have significantly increased following focal seizures in 86% of patients with focal seizures, while it was found to have significantly reduced in 89% of patients with generalized tonic-clonic seizures (GTCS). This finding was interpreted as an indication of a transient change in cardiovascular homeostatic control. 23 In the 2 ^nd^ study, 19 patients with focal seizures and 7 patients with GTCS were investigated. The results of the second study substantiated the results of the first study, in that a substantial decrease was observed in BRS following GTCS. 23 In another study, in which the invasive EEG records of 12 patients were reviewed, administration of intracerebral electrical stimulation revealed that the stimulation of the Brodmann area 25 (BA25) was selectively associated with striking systolic hypotensive changes. 25

Cardiac pathology may also be of interest, given the high prevalence of myocardial fibrosis and myocyte hypertrophy in SUDEP patients. 26 In this context, cardiac echocardiographic biomarkers associated with the risk of sudden death in the general population were studied in 30 patients with temporal lobe epilepsy without history. As a result, significantly higher left ventricular hypertrophy, left ventricular pressure and left atrial volume, and an increased prevalence in at least one of the echocardiographic biomarkers of sudden death was determined in patients with epilepsy compared with the control subjects. 27 Nevertheless, in another study, in which cardiac pathologies of 25 SUDEP cases were compared, the 285 sudden arrhythmic deaths and 104 trauma deaths of non-epileptic patients revealed that the cardiac pathologies of SUDEP cases were similar to those of trauma cases and that the number of sudden arrhythmic deaths was significantly lower among non-epileptic patients. 28

Yang et al. diagnosed a decrease in HRV parameters in 51 patients with resistant epilepsy. It was noted that most differences in HRV values, particularly in the patient group, reached their maximum in the early morning hours, typically around 5 or 6 a.m.. 29

Hawkins et al. investigated differences in heart rate responses to a period of hyperventilation between controls and patients with cardiovascular disease in a retrospective analysis of 9 studies with 282 participants. In their study, it was shown that the increase in heart rate after hyperventilation was statistically significantly reduced in patients with cardiovascular disease. 30

The findings of this study revealed that despite the growing epidemiological data and numerous studies conducted on peri-ictal and interictal physiological factors in patients with epilepsy, the capasity to predict SUDEP risk in individual patients or small populations remains limited. A better recognizing of SUDEP pathophysiology and individual risk assessment is necessary to conduct interventions and studies as well as to enable healthcare professionals to provide more informed counseling to patients regarding their unique SUDEP risks. Studies investigating potential SUDEP biomarkers using the SUDEP-7 inventory as a risk marker are important and should be interpreted with care.

Resting HRV parameters have long been investigated as a predictor of cardiovascular disease and mortality. Studies have shown that HRV may not be consistently abnormal at rest. Therefore, inducible HRV may provide useful information. Hyperventilation-induced heart rate response may provide clinically useful information. There are limited studies in the literature showing a possible association between HRV changes and SUDEP risk in epilepsy patients, but the exact mechanism remains unclear.

In our study, we found statistically significant decreases only in RMSSD, SDRR _HV_ and RMSD _HV_ values in HRV _HV_ parameters during rest. An inverse correlation was found between SUDEP-7 scores and RMSSD (r = 0.578) and RMSSD (r = 0.749) values among HRV parameters. According to the Spearman correlation, SUDEP-7 scores showed a moderate correlation with RMSSD and the strongest and most significant correlation with RMSSD. Other HR parameters (HR, max–min mean HR, SDRR, HRV-TI) did not show significant correlation with resting and post-HV SUDEP-7 scores.

Studies have shown that HRV during rest has its own prognostic value for DRE and does not always yield significant results. 16 These HR changes caused by HV are affected by the interaction between various physiological control systems, especially the autonomic nervous system. 29 30 It has been observed that patients with changes in HR have impaired vagal functions, which probably explains the strongest correlation with RMSSD in DRE patients found in our study.

In conclusion, demonstration of a strong correlation between RMSSD _HV_ values and SUDEP-7 inventory in DRE patients is one of the most important results of our study and has a strong prognostic value. These findings are promising in terms of using the relationship between RMSSD values and SUDEP-7 inventory as a potential biomarker of SUDEP in patients with DRE. Further large-scale investigations are needed to support these findings in terms of their potential utility as a simple, accessible, and inexpensive pre-screening test.
